# Wet Nurse or Milk Bank? Evolution in the Model of Human Lactation: New Challenges for the Islamic Population

**DOI:** 10.3390/ijerph19159742

**Published:** 2022-08-08

**Authors:** Blanca Espina-Jerez, Laura Romera-Álvarez, Mercedes de Dios-Aguado, Aliete Cunha-Oliveira, José Siles-Gonzalez, Sagrario Gómez-Cantarino

**Affiliations:** 1 Department of Nursing, University of Alicante, Carretera de San Vicente del Raspeig s/n, 03690 Alicante, Spain; 2 ENDOCU Research Group (Nursing, Pain and Care), Department of Nursing, Physiotherapy and Occupational Therapy University of Castilla-La Mancha, 45071 Toledo, Spain; 3 Faculty of Physiotherapy and Nursing, Toledo Campus, University of Castilla-La Mancha, 45071 Toledo, Spain; 4 Health Sciences Research Unit: Nursing (UICISA: E), Nursing School of Coimbra (ESEnfC), 3004-011 Coimbra, Portugal

**Keywords:** infant care, neonatal nursing, paediatric nursing, breastfeeding, milk banks, reproductive history, history of nursing

## Abstract

(1) Introduction: The establishment of milk banks in the Islamic world as well as donation to Islamic families in Western countries remains a challenge in the context of human lactation. Religious reservations established since the Qur’an and regulated at the legal–religious and medical level equate milk kinship with consanguinity, which prevents donation. The aim of the study was to analyse the evolution in the model of breastfeeding and care in Islamic society. (2) Methods: The methodology of comparative history was applied, following the structural–dialectical model of care. Historical manuals, articles and databases were analysed. (3) Results: Paediatric care in medical manuals from the 10th–15th centuries is similar to that practiced by the Muslim population today, some beneficial, some harmful; the wet nurse had to follow a series of dietary habits and have a series of physical, moral and educational characteristics in order to be hired. They constituted a beginning of pseudo-professionalisation, in a domestic–family framework. Human milk was used as a remedy for different health problems. (4) Conclusions: Islamic society and nursing have to evolve towards transnational care adapted to the needs of the population.

## 1. Introduction

Despite advances in infant nutrition and technological changes, there is scientific unanimity that breastfeeding during the first months of life is the gold standard [1]. From this framework, other feeding methods beneficial to the health and development of the infant arise [2].

The World Health Organization (WHO) and other international governmental and non-governmental organisations recommend that breastfeeding should be exclusive for at least the first six months of life [3,4]. Considering that its nutritional and immunological properties have not been surpassed to date, the promotion and dissemination of human breastfeeding have been increasing [5,6].

In addition, there are cultural contexts in which human milk takes on meanings that go beyond nutrition and physical health [2,4]. As Margaret Mead pointed out [7], breastfeeding is not only explained by its biological and nutritional component, but also by its cultural symbolic value. The act of breastfeeding is capable of generating imaginaries and representations that construct identities and regulate the way relationships are established. This is a reality that continues to this day in Islamic countries in relation to human breastfeeding, which still includes remedies from traditional food [2,8,9,10]. Therefore, in order to better understand this situation, this article looks back to the Western origins of the intercultural Hispano-Arab territory.

With the Arab conquest of the present-day countries of Spain and Portugal in 711 AD, a territory they called al-Andalus, an intercultural climate arose in which different social groups coexisted. The Andalusians were the native generation of al-Andalus. Moreover, this socio-cultural integration was reflected in the sciences. The Arabs integrated the Greco-Latin and monastic knowledge of conquered Spain with their own medicine with a frequent prophetic tinge, where breastfeeding and the woman who breastfed, whether she was a biological mother or a wet nurse, were protected [11].

However, it is believed that the origin of the figure of the wet nurse can be traced back to human beings living together in groups [12]. The earliest appearances are in the Laws of Ešnunna (1950 BC) in Mesopotamia, the Code of Hammurabi (1750 BC) in Babylon and the Ebers Papyrus (1550 BC) in ancient Egypt [13]. The trade was also recognised by the ancient civilisations of Greece and Rome [14]. Even the Prophet Muhammad was nursed by a Bedouin wet nurse, Ḥalīma, when his mother became ill and was unable to breastfeed him for more than a week [15,16]. The importance that the Qur’an and the Ḥadīz gave to the wet nurse had a great impact on Muslim society at the time and today.

In the Catholic context, with a heritage of seven centuries of Muslim presence (VIII–XV centuries), the figure of the pasiega wet nurse appears, a peasant woman who is hired by the high bourgeoisie, the aristocracy and even the Spanish Royal House to breastfeed and care for children [17,18]. Thus, throughout history, different ways of ensuring breastfeeding and thus infant survival have been observed. Today in the West, the model has been changing. Gradually, the figure of the wet nurse has been in decline, replaced by donation through milk banks. In order to ensure human milk when breast milk is not available, milk banks have been set up and regulated in 60 countries around the world [1], 27 of which are in Europe [19]. However, there are currently no milk banks in Islamic countries. Recently, a process was started in Turkey, but it was abandoned due to its incompatibility with the Qur’anic regulation and the socio-cultural beliefs of the population [20,21,22]. 

The concept of human milk banking (HMB) raises a new debate for contemporary religious people, e.g., does the use of milk create a kinship with donors? Is it the milk itself that creates the biological link, or is it the act of suckling at the breast? There are many unknowns that arise [23], and organisations such as the Islamic Organization for Medical Sciences (IOMS), the International Islamic Fiqh Academy (IIFA) and the Islamic Fiqh Academy (IFA) have attempted to respond through non-binding guidelines or fatwas [24].

In order to understand the current situation, it is useful to go back to the study of the legal–religious and medical tradition of Muslim and Andalusian breastfeeding, and to compare both socio-health situations. Its imprint remains in Islamic societies today, and it is still a little explored subject.

During the X–XV centuries in al-Andalus, there was a whole religious, legal and medical regulation of breastfeeding, which took into account the three essential figures: the mother, the wet nurse and the infant. From the Muslim legal and medical perspective, the wet nurse was widely known. Medical treatises focused on the physical, moral and spiritual characteristics of these women, and on the quality of milk and diet. There is research that deals with the work of women in al-Andalus [25,26,27,28,29,30], but few study the situation of women caregivers and, in particular, the care provided by wet nurses [16,31,32,33]. Moreover, there are few works that take into account the primary sources of medical treatises [25,31]. Therefore, this research aimed to provide new information on the dietary habits of the wet nurse, the different therapeutic properties of milk, the process of breastfeeding and physical and physiological characteristics of the breast and the milk of wet nurses. The aim of the study was to analyse the evolution of the breastfeeding and care model in Islamic society. To this end, the ancestral patterns of care exercised by the Andalusian wet nurse were compared with the care currently practised for the infant. The positions of important health organisations and the limitations existing for Islamic society in the current system of donation through milk banks were taken into account. 

## 2. Materials and Methods

### 2.1. Study Design

The comparative nursing (CN) methodology was used. This methodological framework makes it possible to compare and highlight the similarities and differences between variable care systems, in this case, the same culture in different international settings and different historical moments. The comparative education (CE) methodology studies transnational variations in the field of care, trying to systematise and describe these differences in an attempt to explain them [34]. 

The methodology of CE comes from comparative history, a long-established speciality that is now almost an independent methodology of general history. There are numerous authors who apply the comparative method to history in order to study the evolution of societies (their legislation, norms, beliefs, religions, forms of relationships and care, etc.) [35,36]. 

To respond to the aim of the study, the ancestral patterns of care exercised by the Andalusian wet nurse were compared with the care given to the infant today. The limitations existing for Islamic society in the current system of donation through milk banks were taken into account. 

The variability of this large transnational community was studied by dividing it into different units of analysis using the dialectical structural model of care (DSMC). This model is ideal for studying the social and cultural history of care, which is fundamentally linked to the gender and sexual division of labour [37,38].

**Figure 1:**
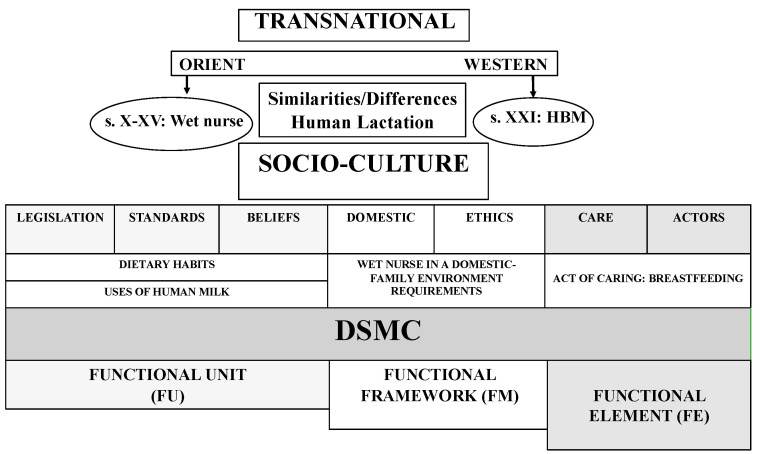
Theoretical dialectical structural model of care (DSMC): application of its structures. Source: authors’ own elaboration.

The DSMC makes it possible to analyse care structures in order to subsequently establish relationships between them. For this study, its application is of utmost importance taking into account the social and cultural aspects that interact in the assistance and care provided by Andalusian wet nurses. The structures used are: (1) functional unit (FU), which represents the social structure of coexistence and socialisation that transmits values, norms, beliefs, knowledge and feelings, through which social systems are constructed, and which determine the sexual and gender division of labour, so decisive in the health and care professions. In this case, it has to do with feeding patterns, care and different uses of milk that are regulated at the medical and legal–religious level, which directly affect the wet nurse and the newborn or infant; (2) functional framework (FF), which refers to the space enabled to develop care activities, specifically, the domestic framework; (3) functional element (FE), which integrates the social actors in charge of care as well as care actions [39]. This research proposes four thematic blocks analysed through the DSMC, each of them representing structures specific to the model of comparative cultural and social history [40]. The four thematic blocks are: (1) dietary habits of the wet nurse and the infant; (2) human milk as a therapeutic remedy; (3) wet nurse in the domestic–family environment: personal and occupational requirements; (4) the process of human lactation and weaning (Figure 1).

### 2.2. Search Strategy and Review Process

The comparative history process begins with an exploratory research question, aimed at synthesising and comparing existing knowledge [34], in this case, “what similarities and differences exist between the wet nurse and infant care in al-Andalus, with respect to today’s Islamic society?”

To answer this question, the DSMC was also applied. The researchers agreed on the eligibility criteria, which had to contain information on the Andalusian wet nurse, from the X to the XV centuries. The review includes documents describing the dietary habits to be followed, the requirements at different levels to be fulfilled, as well as the whole breastfeeding process. In addition, it investigates how the therapeutic and traditional uses of human milk continue to act as a remedy in populations that do not have access to other curative resources. This is compared with the current situation of the Muslim population in Islamic and Western countries. 

The review includes medical treatises from the X–XV centuries, a historical moment of Arab science’s own creation and scientific contribution to the history of medicine and care [41], peer-reviewed articles, formal dissertations, proceedings and reports. The review excludes conference proceedings, proposals and editorials. The documents consulted were in English, Spanish and French. 

The search was carried out between March and July 2021. Several databases were consulted: (1) PubMed; (2) Cochrane; (3) Latin American Health Bibliographic Database (CUIDEN); (4) Scopus; (5) Web of Science; (6) SciELO. In order to have up-to-date information, the database search was limited to the last 10 years.

We also searched the National Historical Archive, the Royal Library of the Escorial Monastery and the Arabic Studies database of the Spanish National Research Council (CSIC). Sources from the Library of the University of Castilla-La Mancha and the Public Library of Toledo were also used. In this case, due to the fact that this is also a historical subject and perhaps not so prone to bibliographical updating, previous publications were consulted and selected because of their interest in the last 10 years. The documentation concerning primary and legislative sources inevitably comes from the period under study.

For the database exploration, a natural or free-text language was used, normalised and controlled with MeSH and DeCs descriptors. These were combined with Boolean operators (“AND/AND”, “OR/OR”, “NOT/NOT”). The results obtained and used, as well as the search equations and filters used to arrive at them, are summarised below (Table 1).

This type of review aims to explore the existing evidence on a particular topic and historical period. The information found is interpreted, juxtaposed and compared, trying to find similarities and differences [34,42]. In this case, dealing with the same culture in different historical moments, with reference to a current socio-health dilemma. By consensus of the authors, articles, medical treatises, books and historical archival documentation were reviewed. A total of 76 documents was reached, following the inclusion and exclusion requirements.

### 2.3. Data Analysis

The documentary analysis was conducted from a qualitative perspective, systematically following the objective of the study. The steps followed in the analysis were: (1) thematic liaison; (2) preliminary classification of the documents based on inclusion and exclusion criteria; (3) selection of relevant information; (4) interpretation and comparison of the results. The selected material was analysed from the point of view of the four thematic blocks of study, each of them encompassed in the structures of the DSMC: (1) dietary habits of the wet nurse and the infant; (2) human milk as a therapeutic remedy; (3) wet nurse in the domestic–family environment: personal and occupational requirements; (4) the process of human lactation and weaning. These blocks were contextualised in the Muslim population of the Andalusian period (X–XV centuries) and the current Muslim population in Islamic and non-Islamic countries. To extract and summarise the data, the first and second authors carried out a general data extraction. The third author examined the findings in depth. The fourth and fifth authors identified the thematic blocks encompassed in the DSMC structures, from functional unit, functional framework and functional element. Discrepancies were resolved by consensus among the researchers. 

## 3. Results

### 3.1. Dietary Habits of the Wet Nurse and Shared Recommendations Regarding Disease

The dietary recommendations generally indicated that wet nurses should be restrained with food. They should not eat salty, spicy, acidic or highly seasoned foods. Averroes [43] gives leek, onion, garlic and celery as examples. Medical justification is based on the balance of humours [44]. For this reason, the medicines intended to make the “milk flow” should be those which “warm the phlegmatic humours and assist the digestive power of the organs. There are more effective foods, and they are those which engender a chyme balanced in warmth and moisture, similar to the warmth and moisture of the blood” [43].

The recommendations to be followed by the wet nurse revolved around the following products: broad bean flour, rice, dried semolina bread, sugar, fresh fish and well-cooked meat, and if spices were used, they should be few and with special consideration of fennel seeds [26]. Likewise, the foods most likely to benefit the production of breast milk were wheat-based foods, goat and lamb meat, eggs, almonds and lettuce [45]. When the wet nurse was not fed according to these guidelines, the milk could become impure and affect the child’s health and character [26,32,43,44,45].

There was a very close, even symbiotic, relationship between the child and these women. Thus, when the child fell ill, the wet nurse had to modify her diet in the first instance. When this did not work, the doctor would indicate dietary guidelines for both of them. Sometimes these could be different, sometimes similar (Table 2).

A *yawaris* or *yuwāriš* is an electuary, or pharmaceutical, preparation made from various ingredients, almost always vegetable, on a sugary base, which in this case results in the mixture of honey or water and sugar with the active medicinal agents. The final preparation was administered orally [46]. 

The dirham is a unit of measurement of weight [41]. The Pillow Book lists this unit of measurement with its gram equivalents. 

### 3.2. The Socio-Health Value of Human Milk: Milk as an Ingredient for Other Ailments

Human milk is a folk ingredient applied in remedies in traditional medicine, natural pharmacopoeia and ethnomedicine. Averroes [43] already indicated that all mammalian milks were good for treating certain ailments, but he stressed that women’s milk was the best. 

The properties of human milk for treating eye problems were well known. Arab medicine, and specifically Andalusian medicine, gave considerable impetus to the field of ophthalmology. Even Dioscorides himself [47] reported that human milk, mixed with crushed frankincense, should be instilled into bleeding eyes that have suffered trauma. Additionally, Ibn Said [44] recognised the importance of human milk for ophthalmic treatment. He stated that no medicine should be applied to the eyes because of their sensitivity, so they should be cured with freshly expressed milk or egg white. Various practitioners of the time treated such eye complaints (Table 3).

However, human milk also appears as an ingredient to treat other ailments. At the otic level, it was recommended to facilitate the removal of plugs in the ears and to cure their inflammation [48]. Likewise, Ibn al-Baytar [49] recommended taking opium combined with women’s milk and saffron for the treatment of gout.

In the paediatric field, breast milk became particularly important for treating common illnesses. For children’s coughs, it was recommended that Arabic water, white traganto, liquorice extract and feverfew be mixed and then dissolved in human milk. In the case of mucosal ulcers, especially mouth, uterine and anal ulcers, it had analgesic functions and also could be combined with analgesic medicines. Finally, it was also used to treat feverish conditions [44].

The effect of human milk for the treatment of dry eye syndrome has now been tested in an animal model. The results of a study showed that animals treated with human milk improved in the same way as those treated with conventional cyclosporine therapy. The improvement in those treated with human milk began to manifest itself within 4 days of treatment, as did the antibiotic used [50]. 

Similarly, the use of breast milk has now been documented in Polish women for the treatment of mucosal infections in children. Polish mothers frequently use it against rhinorrhoea, lacrimal obstruction and conjunctivitis. The same authors have documented the use of human milk, also in Polish women, for the care of cracked nipples, and even for the treatment of dermatitis in the nappy area and neonatal acne. For these problems, 58% of women reported having used this method at least once [52,53,54,55,56]. 

Other studies have found a consensus between ancient texts and current literature on the efficacy of topical application of human milk (THM) in paediatric inflammatory problems. Its purposes include curing the umbilical cord [53,54,55,56], atopic dermatitis, napkin dermatitis [56,57,58,59], conjunctivitis [60], dry eye syndrome, scratches, insect bites, perineal ulcers and nipple sores [56]. In fact, there was agreement among the authors of the different studies reviewed. They concluded that it is an effective, safe and widely available treatment compared to other conventional chemical remedies, especially in areas where mothers do not have access to institutionalised medicine [61]. 

### 3.3. The Wet Nurse in the Domestic–Family Environment: Personal and Occupational Requirements

Women’s jobs were carried out in the domestic environment. It was common for the wet nurse to work alongside the Andalusian midwife. In fact, the midwife often assessed the state of health and suitability of the future wet nurse. She also made home visits after childbirth, which had a health education function. The wet nurse was supported by the midwife, who tried to make sure that breastfeeding was going well, or if not, she would indicate guidelines for improvement [30,62]. In fact, Ibn Jaldum [63] indicated that wet nurses and midwives were the ones who best understood and knew how to care for the ailments of infants, even better than the most competent doctors. 

The office of wet nursing was rated as one of the most highly regarded legal jobs of the time, in the female context [64]. They were often women who lived in rural areas and came from low-income families. They could also be educated women, although this was a more exceptional situation. They were hired by middle-income or privileged families to help the mother raise the newborn or to take care of the newborn almost entirely [25].

Breastfeeding and care were established under a contract for the lease of services or iyarat al-a’mal, rather than a lease of material goods or iyarat al-a’yan, such as milk itself. The hiring of wet nurses and the wages they received were legally justified on the basis of general care of newborns and infants during and after the nursing period [26].

The wet nurse had to meet a number of physical, civil and moral requirements. In addition, she had certain restrictions on diet, exercise and sexuality. With regard to physical condition, ‘Arīb Ibn Sa’īd [44] states that she had to be a “young woman between 20 and 30 years of age, of a clean colour between white and red hair, who is not pregnant”, as the milk corrupts and turns into water and nourishes the foetus in the womb, which would cause the pregnancy to be interrupted due to lack of nourishment. Other authors limit themselves to specifying that the dam should have a healthy colour, medium weight and good figure, proportionate in hardness and softness, strong neck and large chest [65]. 

Additionally, the fact that the woman had had several children was a positive factor, as it was considered that the milk would be of better quality, in addition to her own expertise linked to experience. She also had to be free of any illness, either physical or psychological [44]. However, it was not only this physician, ‘Arīb Ibn Sa’īd, who showed concern throughout the work for the negative states of mind of wet nurse and child. Averroes also [43] repeatedly indicated in his Book of the Generalities of Medicine that it is desirable for the wet nurse to avoid circumstances that would distress the children and thus alter their complexions. In the same vein, Ibn al-Khatib also [45] noted that moods of anger, fear and worry were to be avoided in order to avoid their behavioural correlates in the baby. 

As for physical exercise, wet nurses were not allowed to exercise intensively or with any frequency, but rather were expected to lead a sedentary and child-centred life. There is some controversy about sexual relations. On the one hand, it is stated that coitus could not be practised during the days of menstruation [44]. However, such a recommendation was common in the Islamic world, as menstrual blood was considered impure and sexual encounters should not take place on these days [66,67]. 

Physicians, jurists and religious scholars were concerned about the simultaneity of sexual intercourse and human lactation. It was thought that intercourse could affect human milk, threatening the health of the infant. Physicians recommended that all sexual activity should be suspended during this period, arguing that the odour and properties of the milk would be altered [43,62]. The most popular prophetic traditions held contradictory views. The school of Mâlik permitted sex while breastfeeding, but the schools of Abu Dawud and Ibn Maga claimed that it was harmful, as it morally corrupted the milk, and should therefore be forbidden [68,69]. Given the general agreement on sexual restraint, the wet nurse’s husband had to authorise it, and the signing of the contract committed them to the specified sexual abstinence clause [62,70]. On the other hand, given that lactation was to last for two years, it is possible that sexual restraint was one of the main reasons for the hiring of wet nurses.

### 3.4. The Act of Caring through Breastfeeding

#### 3.4.1. A Journey: From the Beginnings of Human Lactation to Weaning

After sprinkling and massaging the baby with salt, the midwife would hand it over to the breastfeeding mother or wet nurse. On this point, there is some disagreement among physicians of the time. For Ibn al-Khatib [45], the mother’s milk was the best, as it was somehow understood that there was already an implicit and physical knowledge between mother and child, and the mother’s nipple had more and better qualities to avoid any harm [45,65]. However, ‘Arīb Ibn Sa’īd [44] believed that after birth, the baby would drink the milk of a woman other than the mother for at least four days. This unphysiological procedure was recommended because it was believed that colostrum was an impure milk, which instead of nourishing purged the infant [71]. Ali ibn-al-Abbas al-Majusi [72] recommended feeding the newborn baby in its first two days of life with sugar crushed in sesame oil. Recent ethnographic studies show that this practice continues today. Some mothers even avoid breastfeeding newborns for the first two days of life and discard colostrum. They claim that the milk is expired or impure. Honey, fenugreek, butter, water and aniseed were and are used today as substitute foods in cases where it is decided to feed by creating a pre-milk stage [2].

A very paradoxical aspect, if we consider the current pattern in breastfeeding, is that the frequency of feedings had to go from less to more. Medical recommendations suggested that in the beginning, two or three feedings a day were thought to be sufficient, and in order to achieve this, breastfeeding was supplemented with food substitutes such as honey. If the infant cried and was unable to sleep, then it was breastfed [65]. Ibn Sa’īd, however, did not believe this to be sufficient [44] and recognised the importance of the infant being well nourished, finding a balance between remaining satiated and not overeating, otherwise gastric distension would lead to vomiting. If this were to occur frequently, feedings would be spaced out, with smaller amounts given and gradually increased, according to tolerance. Therefore, this doctor was in favour of what is known today as demand feeding. However, if vomiting was very frequent, remedies were recommended both for the infant and for the wet nurse.

Averroes [43] and Ibn al-Jatib considered that children should be exclusively breastfed until their teeth came in, specifically the incisors. At that time, they would gradually begin to eat “soft” foods and would be helped in the eruption of the incipient teeth by feeding hare’s brains and hen’s fat [43,45]. Starter foods included acorns of semolina flour, cow’s milk, mild soups or crumbs with broth, sugar and chicken breast meat or partridge [44,65]. 

There are references in the Qur’an to the timing of weaning. Specifically, it indicates that the timing can be agreed upon between the father and the woman [67]. ‘Arīb Ibn Sa’īd [44] did not specify much more about when to switch to the combination of breastfeeding with food, but he did set out two key points. The first is that breastfeeding should last for a full two years, and could be extended to thirty months, as the Qur’an puts it [73,74]. Secondly, that weaning should not take place during the summer, as it increased the risk of gastroenteritis [44]. 

Currently, the Global Strategy for Infant and Young Child Feeding recommends that infants should be fed human milk for the first six months for optimal growth, development and health. During this period, the infant should not be given any other food or drink except rehydration salts, vitamins, minerals or appropriate medication. After the first six months, they should receive complementary foods without abandoning breastfeeding until they are two years of age or older [75]. 

The WHO and the Spanish Association of Paediatrics do not establish a specific time for weaning, leaving the decision in the hands of the mother and the infant. It is advisable to do so gradually, without offering or withholding the breast, for example, by negotiating certain places or specific situations with the child. At this stage, it is important to look for alternatives to the need for contact between the two, given that the bond established through breastfeeding is very close, and must be progressively reoriented [75,76]. In contrast, the Muslim population recognises two weaning stages (Table 4).

If it was difficult for the child to move on to the second stage, mu’affar, and the reason was that he or she longed for the mother’s breast, “little balls made of bread and sugar” were given to the child [65]. However, if the desire to suckle remained strong, a bitter and unpleasant smelling ointment, such as “aleña or similar repellent substances”, was applied to the nipple [65]. 

#### 3.4.2. Wet Nurse: Breast Characteristics and Milk States

The mammary requirements specified by ‘Arīb Ibn Sa’īd [44] included having a broad thorax and a well-developed breast, a feature that coincided with Ibn al-Khatib [65]. In addition, she should have medium-sized nipples, as larger nipples may hinder breastfeeding [44]. 

The milk should be white, aromatic, not too fluid, not too thick, with not too much cream [44]. There was a way of testing the texture and fluidity of the milk. In this respect, Ibn al-Khatib [65] considered that if the milk came out with impetus, it was because it was watered down; instead, when the opposite happened, the milk was thicker than it should be. Likewise, to find out the liquid concentration of this substance, milk was added to a glass with a little brine and stirred with the fingers. Therefore, when this liquid was affected, the doctors collected a large number of remedies depending on what the alteration was [65] (Table 5).

Today, in the Western world, the figure of the wet nurse has evolved towards the increasing establishment of HMB [1,19]. However, the incompatibility with legal–religious reservations prevents the implementation of HMBs as they are currently understood in the West [20,21]. Only a few cases are known of pre-term babies who have received donated human milk, in these cases the families have met and agreed on religious reservations [23].

In fact, in the study conducted by Karadag et al. [21] with 1042 Muslim mothers, it is observed that most of the mothers interviewed are against the establishment of Western-style milk banks. However, they show a positive response to an alternative milk bank that is able to preserve their religious concerns. In the study by Onat and Karakoç [22], the starting rules would involve knowing the gender of the baby, limiting donors to three, and knowing the identity of the donor and recipient so that “milk kinship” can be safeguarded. Thus, 77.3% of the Turkish participants said that there was a need to establish an HMB in Turkey. 

## 4. Discussion

Social and cultural history, in a comparative framework, has to be considered as a dynamic and global science. It offers a synthetic and juxtaposed vision of the same society at different historical moments. In the field of this study, it also makes it possible to analyse the care of the same culture through a different space–time. 

It also shows how Islamic medicine focused its attention on the bodily and spiritual health of the wet nurse, as well as the balance between the two, which would bring about the good health of the child. Through breastfeeding, wet nurse and infant are constituted as an inseparable unit, so that when the infant becomes ill, the physician must assess the condition of the wet nurse. A search should be made to see if there is any physical or psychological cause for the infant’s illness, and if so, the infant’s feeding should be changed or the appropriate imbalance corrected [41,43,44].

It is worth noting the presence of wine and other alcoholic beverages as an ingredient in some of the preparations and recipes analysed in this study. It is well known that alcohol was forbidden by Islamic law. However, this issue is a point of controversy [77,78] deduces the existence of a double standard that allows certain groups to remain outside of the requirements. These include people with economic privilege and healing needs. Regarding medicine, Ibn al-Khatib [45] introduced it as an ingredient in recipes for vulnerable age groups, the elderly and children, and prescribed it to people of a cold complexion and in depressive moods. 

The biological roles of the wet nurse were centred around breastfeeding, and took place within the family household that required her services. In contrast to the Christian wet nurse [17], only a few Muslim wet nurses are known to have nursed important historical figures [62]. The fact that doctors and religious scholars agreed that breastfeeding and sexual relations could not coexist made it impossible for women to continue procreating. For this reason, one of the vital clauses in the contract for both the Andalusian and Christian wet nurse was to prevent an active life, let alone a sexual life, due to believing that the health of the infant and the quality of the milk would be corrupted [43,62,68,69]. In this way, they served the function of enabling the new mother to recover as soon as possible, have sex before the two years of compulsory breastfeeding and become a mother again. 

Furthermore, this research attempts to compare the ancestral patterns of care exercised by the Andalusian wet nurse with the care practised by Muslim women today in some points, such as disposing of colostrum and introducing pre-milk feeding, frequency of feedings, combined feeding or the total duration of the breastfeeding period. At present, it highlights the need to adapt the breastfeeding framework, given the reduction in informal milk exchange through the wet nurse, and the difficulties of moving towards a model of milk donation, due to religion reservations. Informal milk donation also runs the risk of disease transmission if certain measures are not taken [22]. 

The question is not whether breastfeeding, milk from a milk bank or that from a wet nurse is better, but to analyse the conditions in which these feeding practices are given to children. In other words, the health needs of a particular society or group can only be understood within the socio-cultural context. The important thing is to understand a problem or a special situation from the inside and how it is perceived by those who are involved in it [3]. In addition, currently, a part of the Muslim society affirms that it would accept the implementation of milk banks as long as religious reservations are maintained. For example, as the women in the study by Onat and Karakoç [22] indicate, knowing the sex of the baby, limiting the donation to a maximum of three babies (to reduce the risk of marriage when a relationship already exists), and knowing the identity of the donor and recipient.

On the other hand, recognising these ancestral practices allows for the conservation of the most useful and scientifically proven practices. Especially for those environments where the socio-economic and socio-health situation makes access to unanimously approved therapies difficult, they can be decisive for child recovery and survival [61]. For example, the use of human milk for the treatment of different ophthalmic, otic and different paediatric pathologies has proven to be effective in the same way as conventional Western therapies [50,51,52,53,54,55,56,57,58,59,60]. It is also interesting to note that there is no reference in the medicinal recipe books to the religious controversy that might arise from the donation of milk as a single ingredient or as part of a set of ingredients for a cure. Religious scholars must therefore ask, what is it that really generates the kinship of milk between donor and recipient? 

Intervention from an institutional framework that is respectful and non-aggressive towards the culture, through health education, would make it possible to approach the conservation of practices that have proven to be effective, and the reconsideration of those practices that are harmful to the newborn or infant. For example, the belief that colostrum is an impure milk that has to be replaced by the aforementioned foods, creating the pre-milk stage or the frequency of breastfeeding that goes from less to more, when paradoxically the opposite is true in demand feeding [2,44].

## 5. Conclusions

This work reflects how the family has historically constituted the basic institution that has provided health care, with women being specifically responsible for breastfeeding, upbringing, growth and education, through the sexual division of labour. Despite the importance given by Islam to the figure of the wet nurse, due to her relevant function at the socio-health level, few paediatric sources and treatises have been written and survive to the present day. From the 10th–15th century, the sources reveal a domestic–family model of care through the hiring of wet nurses. In other words, these are the beginnings of the pseudo-professionalisation of nursing and child-rearing, which later acquired a similar model in Christian society.

However, the scarcity of sources on the professionalisation of the wet nurse’s profession is an important limitation. Legal sources provide information on the rights, duties and limits of the wet nurse. Medical sources provide an overview of paediatric, well and sick child care. However, it is difficult to know what happened within the family–domestic environment in which the work was carried out. This may have been due, among other factors, to the fact that women in the 10th–15th centuries were forbidden to write books or treatises on their care.

In any case, the permanence of Andalusian science and its study in medical schools and universities continued in the Western and Eastern world after a few centuries. The trend towards the disappearance of the wet nurse in the West has been driven by scientific, industrial and political advances. However, in the East, many of the practices of traditional medicine found in the medical treatises studied are still active. Muslim societies in the East and West have not evolved in the same direction and are facing ethical and moral dilemmas that pose new challenges in the breastfeeding–nursing process. 

Institutions and professionals must continue their efforts to maintain an attitude of predisposition towards the emic vision of the social groups present in the health care field. Care, and nursing in particular, should be approached from a framework of increasingly integrating cultural competence, which favours the humanisation of care through intercultural coexistence within and outside transnational health systems.

## Figures and Tables

**Table 1:** Thematic blocks related to references.

Database	Search Strategy	Filters	Points Extracted	References
Pubmed Cochrane Cuiden Scopus Web of Science SciELO Books Treatises	breastfeeding AND human milk AND wet nurse AND muslim wet nursing AND muslim history wet nursing AND requirements AND muslim history wet nursing AND milk siblingship AND muslim history wet nursing AND legislation AND muslim human milk AND remedies breastfeeding AND child survival AND (muslim) breastfeeding AND Quran nursing mother AND Quran nursing mother AND traditional medicine wet-nursing AND Middle Ages wet-nursing AND nursing history breastfeeding AND colostrum AND muslim mothers	Last 10 years Article English/Spanish	Dietary habits of wet nurse and infant Therapeutic uses of human milk Domestic–family work. Requirements of the wet nurse Breastfeeding and weaning. Breast and milk characteristics	[26,32,41,43,44,45,46] [41,43,44,46,47,48,49,50,51,52,53,54,55,56,57,58,59,60,61] [25,26,30,43,44,45,61,62,63,64,65,66,67,68,69,70] [2,43,44,45,64,65,66,70,71,72,73,74,75,76]

Source: authors’ own elaboration.

**Table 2:** Common diseases of the infant. Dietary recommendations for the wet nurse and infant.

Infant Disease	Feeding of the Wet Nurse	Infant Feeding
Gastroenteritis	Lamb roasted over charcoal fire, previously sprinkled with rose water and quince water. Bread made of wheat and millet flour in different quantities.	A roasted Armenian clay *ratl*, macerated in a quarter of fresh water. If the infant was thirsty, he/she would be given this water to drink. The clay would act as a filter. A quince *yawaris*, which was dissolved in rose syrup.
Vomiting and diarrhoea	Ten dirhams of roasted Armenian clay with a quarter of water. Eight dirhams of apple *yawaris*, one habba of musk and partridge meat mixed with quince and rose water.	

Source: authors’ own elaboration based on Ibn Wafid [41].

**Table 3:** Aliments and ophthalmic remedies made from mother’s milk.

Source References	Ailment	Remedies
Ibn Wafid (10th c.) [41] The Pillow Book	Ophthalmia or ocular inflammation that afflicts children.	Separately, starch is put in rose water until it dries. On the other hand, sarcocola is macerated in women’s milk and left to dry. When both preparations are dry, they are pulverised and an equal quantity of both is added to the eye.
	Dry eye syndrome.	Six or seven times with milk from healthy, breastfeeding women.
	Strengthening the eye and sharpening eyesight.	Mother’s milk combined with honey and instilled with a few drops of vinegar.
Abu Zuhr (s. XII) [48] The Book of Medical Experiences	Ophthalmic eye drops.	Woman’s milk or rose water are used as basic diluents for any eye drops.
	Abundant optic discharge.	Nightly application of egg white or almond milk mashed with women’s milk on the eyelid.
	Removal of a foreign body, ocular and eyelid oedema, as well as the onset of a pterygium.	It is used as an ingredient in eye drops to treat these aliments.

Source: authors’ own elaboration based on Ibn Wafid and Abu Zuhr.

**Table 4:** Stages of weaning stipulated in the Muslim population.

Fase	Descripción
(1) *ayat-hu bi-rayyatin wa huwa ‘ayyi*	The exact translation is “rearing without mother’s milk”. This phase occurs when weaning occurs prematurely.
(2) *al-ta’fir*	Feeding is combined with periods of breastfeeding.
(3) *mu’affar*	Child stops breastfeeding

Source: authors’ own elaboration based on ‘Arīb Ibn Sa’īd [44].

**Table 5:** Remedies for optimising breast milk condition.

Milk Status	Natural Remedy
Reducing its thickness	The wet nurse had to drink a lot of water.
Thick milk	Oxymiel: a beverage made up of a mild wine, water and honey.
Very fluid milk	Women would eat foods such as rice, meat and egg yolks, or they would simply have to eat more and heartier foods.
Low quantity	The wet nurse drank water with bran and fennel seeds, salted fish heads and wine.
Low quantity	This was a matter of great concern, the specific aetiological factor would have to be identified in order to apply an appropriate pharmacological treatment, although some electuaries were also recommended.

Source: own elaboration based on de ‘Arīb Ibn Sa’īd [44] and Ibn al-Jatib [65].

## Abbreviations

DSMC	Dialectical structural model of care
FU	Functional unit
FF	Functional framework
EF	Functional element
HMB	Human milk bank
